# Cowden syndrome is a risk factor for multiple neoplasm: a case report

**DOI:** 10.1186/s12957-020-01971-z

**Published:** 2020-08-17

**Authors:** Sofia Miguelote, Roberto Silva, J. L. Fougo, L. E. Barbosa, J. P. Araújo Teixeira

**Affiliations:** 1 Faculty of Medicine, University of Porto, Porto, Portugal; 2 University Hospital Center of S. João, Porto, Portugal; 3 Pathological Anatomy Department, University Hospital Center of S. João, Porto, Portugal; 4 Breast Center, University Hospital Center of S. João, Porto, Portugal; 5 Surgery Department, University Hospital Center of S. João, Porto, Portugal

**Keywords:** Cowden’s syndrome, *PTEN*, Papillary carcinoma, Breast sarcoma

## Abstract

**Background:**

Cowden’s syndrome is an autosomal dominant disease with variable penetrance, involving the tumor suppressor phosphatase and tension homolog gene, located on chromosome 10q22-23, responsible for cell proliferation, migration, and cellular apoptosis. Its clinical presentation encompasses mucocutaneous lesions, which are present around 99% of the time; macrocephaly; and cognitive impairment, and it precedes the appearance of neoplasms such as thyroid carcinoma, breast cancer, among others.

In addition to these malformations, arteriovenous malformations of the brain and spine, endocrine abnormalities, skeletal defects, and cardiopulmonary lesions may also be found.

The relevance of the case is due to the fact that, through a certain phenotype, the patient’s genotype can be inferred and thus followed up closely.

**Case representation:**

The clinical case concerns a 28-year-old Caucasian and Portuguese woman with palmar pits, macrocephaly, and cognitive impairment. She was diagnosed with papillary thyroid carcinoma at 22 years of age and proposed total thyroidectomy.

At age 27, a pregnancy was diagnosed with a Breast Imaging-Reporting and Data System 2-rated breast lump. After the histological verification, it was concluded that it was a high metastatic breast sarcoma, opting for palliative mastectomy. A genetic evaluation confirmed alteration in the phosphatase and tension homolog gene, confirming Cowden’s syndrome. The patient died at age 29 due to neoplastic pathology.

**Conclusion:**

This report aims to alert to the clinical signs of this entity and the clinical supervision and follow-up of these patients. In order to prevent premature deaths and to improve patient’s quality of life, genetic diseases with cancer impact should be diagnosed as early as possible.

## Background

Cowden’s syndrome (CS) was first described by Lloyd and Dennis in 1963, in a 20-year-old patient, Rachel Cowden, who had multiple deformations such as scrotal tongue syndrome, papillomatous papules, thyroid adenomas, fibrocystic breast disease with malignant degeneration, central nervous system changes, and family members with a mild form of the disease [1].

Only in 1996 was the susceptible zone of SC 10q22-23 mapped [2] and the tumor suppressor gene phosphatase and tension homolog (*PTEN*) that regulates the PI3K/Akt/mTOR pathway was identified, which is responsible for the proliferation, migration, and cellular apoptosis [3].

PTEN hamartoma tumor syndrome (PHTS), which comprises CS, adult Lhermitte-Duclos disease (LDD), Bannayan-Riley-Ruvalcaba syndrome (BRRS), and Proteus-like syndrome, represents a spectrum of hamartomatous overgrowth manifestations associated with germline mutations in the PTEN gene [4].

LDD is a rare, slow-growing hamartoma which is usually diagnosed when patients are in their twenties or thirties. While it appears to be clearly associated with PTEN mutations, the incidence of LDD in patients with CS is unknown. Vascular (venous or arterial) anomalies are reportedly common in BRRS and CS, particularly in patients with a BRRS diagnosis. A number of case reports support the association of arteriovenous malformations in clinically diagnosed BRRS patients [5].

It is important to recognize that in addition to LLD, other intracranial findings such as multiple venous anomalies, meningiomas, greater than expected white matter signal abnormality, prominent perivascular spaces, and cortical malformations may warrant a thorough evaluation for CS in the appropriate clinical setting [6, 7].

BRRS is typically diagnosed in childhood and is characterized by macrocephaly, hamartomas (including lipomas or intestinal polyps), penile freckling in males, and developmental delays, including an increased risk of autism spectrum disorder (ASD) [4]. Other reported features include developmental delay, vascular anomalies, large birth weight, and joint hyperextensibility [5].

CS is an autosomal dominant genodermatosis with variable expressiveness, with over 300 germline mutations in the PTEN gene already described but no significant correlation between mutation type and tumor types, and in 20% of the patients, no mutations were found [8]. Mucocutaneous lesions including trichilemmoma, acral keratosis, and oral papillomatosis, plus macrocephaly, are the most frequent features, described in more than 90% of cases after the third decade [9].

Multiple extra-mucocutaneous manifestations can also occur in CS. The skeletal system may form a high-arched palate, scoliosis, or macrocephaly [10].

The estimated prevalence is 1/250,000, with a slight predominance in females and Caucasians and penetrance of up to 90% in the second decade [11]. The diagnosis, normally, is based on clinical criteria, periodically updated by the National Comprehensive Cancer Network® (NCNN®) [12], though confirmatory genetic testing is frequently used.

## Case representation section

The case being studied was reported at the São João Hospital, which is part of the Porto Medical School, University of Porto, in the North of Portugal.

Patient, female, Caucasian, born on 2 September 1989, having concluded year 9 and unemployed at the time.

Family history, mother with oligophrenia and maternal aunt died of thyroid carcinoma.

Personal history of childhood hydrocephalus, menarche at 12 years with irregular catamenia that have been corrected after the introduction of oral contraceptive. In 2008, the patient was directed to psychiatry for suspected cognitive impairment.

In 2010, she had severe microcytic and hypochromic anemia and was treated with saccharized ferric oxide. Gynecological ultrasound without major changes and occult blood in the stool was negative. After hematological research, clinical research von Willebrand’s disease was excluded.

In 2012, she was admitted to endocrinology due to changes in thyroid analytical parameters, TSH and T_4_ and 5-cm thyroid nodule in the left lobe. An ultrasound was performed which identifies a 5.6-cm hypoechogenic nodule and anecogenic areas, the largest with < 1 cm. Subsequently, she performed a fine-needle biopsy and was diagnosed with follicular lesion of undetermined significance (FLUS) (Bethesda category III). After a multidisciplinary meeting, it was decided to intervene the patient with total thyroidectomy.

**Fig. 1:**
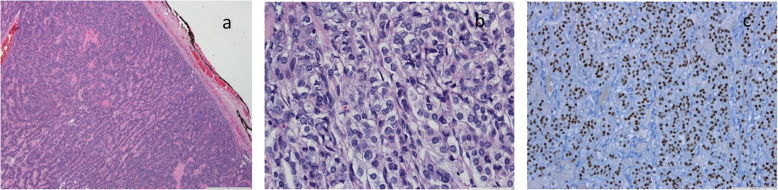
Solid/trabecular variant of papillary carcinoma. Well-defined tumor with trabecular pattern (**a**) composed by follicular cells with nuclear features of papillary carcinoma (**b**) and expression of TTF-1 (**c**)

In September 2012, she underwent 51 g total thyroidectomy and was diagnosed with papillary carcinoma solid/trabecular variant (Fig. 1). Concomitantly, she performs ablative therapy with 3700 MBq (100 mCi) of I^131^ and is treated with levothyroxine and calcium.

In January 2013, research scintigraphy after 24-h oral administration of 18.5 MBq (50 mCi) of I^131^ was conducted, revealing anterior cervical uptake focus compatible with functioning thyroid tissue and no other pathological fixation foci.

After 1 year, in routine ultrasound, microcalcifications were detected in the jugulocarotid chain, possibly related to a secondary injury to papillary thyroid carcinoma.

In November 2017, she was referred to the center of the breast by right breast lump in the upper left quadrant. At that time, she was 38 weeks pregnant and was being followed in obstetrics with a diagnosis of hydramnios, fetal hypothyroidism, and suspected fetal macrosomia opting for childbirth this week.

The breast ultrasound identified a well-circumscribed 3-cm polylobular nodule classified as Breast Imaging-Reporting and Data System (BIRADS-2).

**Fig. 2:**
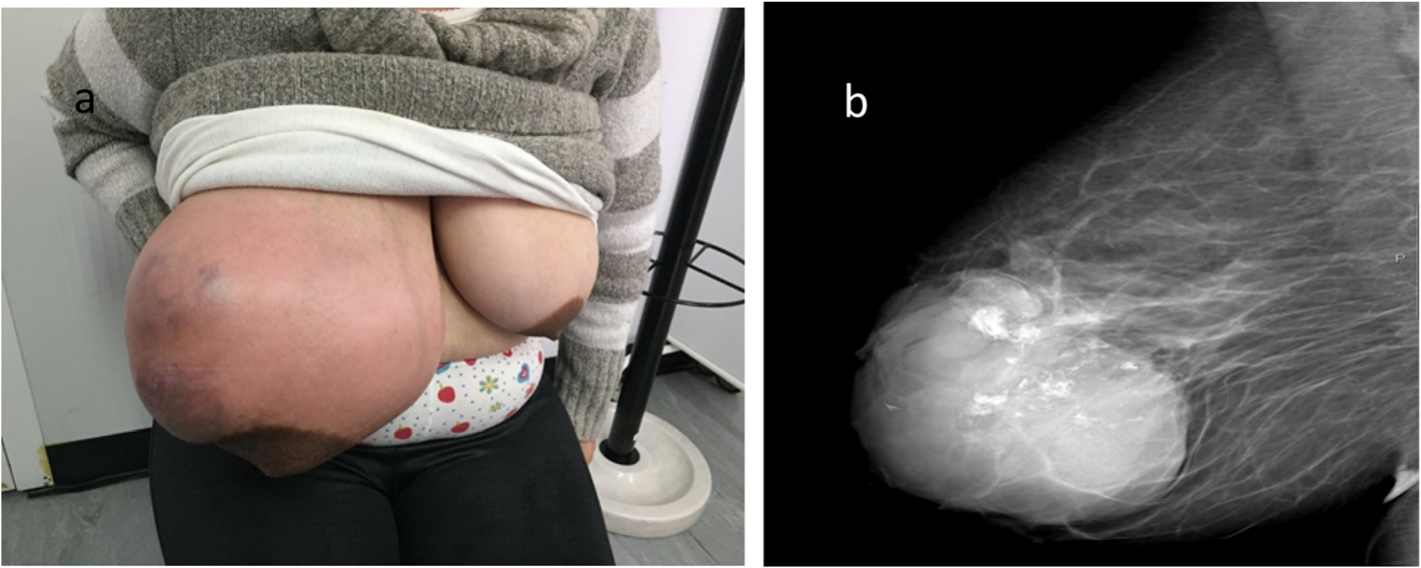
**a** Right breast lump. **b** Radiological image of the breast lesion

In May 2018, the breast ultrasound revealed a 4-cm nodular lesion and FNB was performed. The result revealed a heterogeneous mass of about 10 cm classifying as BIRADS-4C. Mammary computed tomography showed multilobulated contours with gross calcifications (Fig. 2).

**Fig. 3:**
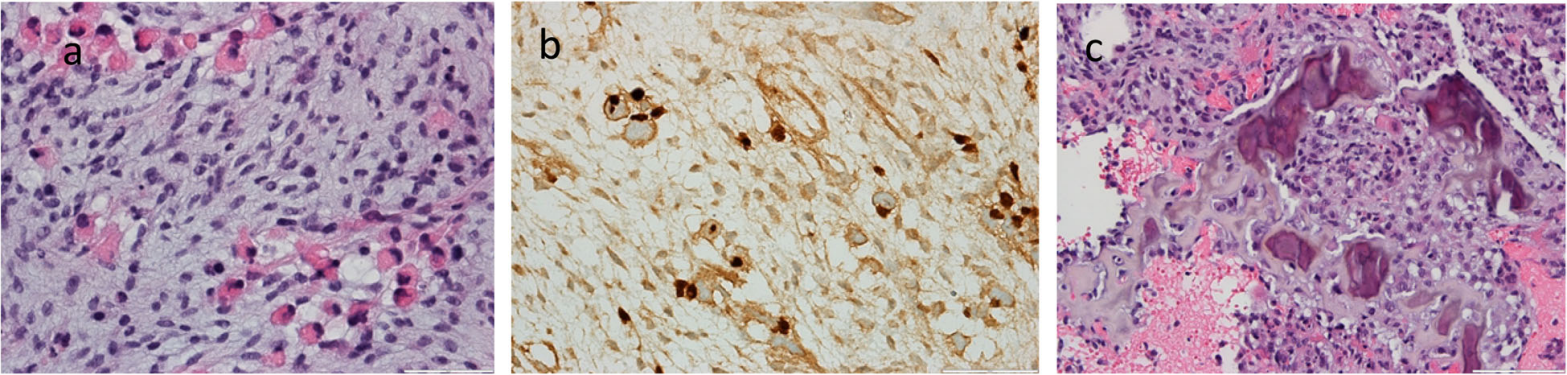
High-grade sarcoma of the right breast composed by atypical spindle cells with rhabdomyoblastic (**a**, **b**) and osteosarcoma (**c**) components

In the histological study of the microbiopsy of the breast lesion, a malignant neoplasm compatible with high-grade sarcoma with areas of necrosis was observed. The neoplasm consists of spindle cells, atypical and with frequent figures of mitosis. The presence of osteosarcoma and rhabdomyosarcoma components is shown in Fig. 3.

It was proposed to the patient to undergo palliative right total mastectomy, and the recommended therapy was doxorubicin and ifosfamide. After explaining the precautions and warnings of the drugs, the patient chose to take doxorubicin only.

**Fig. 4:**
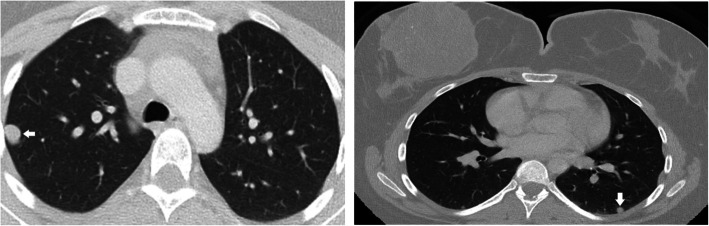
Computed axial tomography with evidence of metastasis (white arrow)

In August 2018, she performs bone scintigraphy revealing no image suggestive of focal metastatic and/or tumoral bone pathology with hyperfixation in right breast dependence compatible with heterotopic fixation by the breast lesion studied. For staging, she underwent computed tomography where nodules suggestive of metastases were found in the different lobes of both lung fields, all with a diameter less than 1 cm (Fig. 4).

**Fig. 5:**
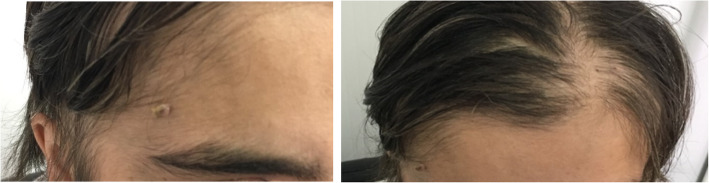
Mucocutaneous lesion on the face and scalp

Concomitantly, she was admitted for a genetic consultation, showing clinical macrocephaly (58.1 cm), round keratotic palmar pits, and wart vulgaris on the face and scalp (Fig. 5).

The genetic evaluation included the genes *PTEN*, *TP53*, *BRCA1*, *BRCA2*, *ATM*, *CHEK2*, and *PALB2*. *PTEN* gene alteration was found. The result coincides with a change in heterozygote, pathogenic variant c.405dupA (p. (Cys136Metfs * 44) in the *PTEN* gene. This variant leads to the formation of a premature stop codon and potentially the appearance of a truncated protein.

She has 3 stepbrothers and 1 stepsister, but she has no relationship with them. The mother and uncles do not want to carry out the genetic test, and the siblings are unreachable. Genetic tests were not performed on the patient’s limbs because they do not want to know.

In 2019, she died due to extensively metastatic breast cancer (Table 1). Since the diagnosis of papillary carcinoma at her death, only 11 years have passed, which demonstrates that the diagnosis made correctly and the time could have dictated another path.

**Table 1:** Clinical condition summary

ANO	Idade	DIAGNÓSTICO
2008	19	Oligophrenia
2010	21	Microcytic and hypochromic anemia
2012	23	Papillary thyroid carcinoma
2017	28	Breast sarcoma
2018	28	Cowden’s syndrome diagnose Sarcoma metastasis
2019	29	Death

## Discussion and conclusion

CS presents itself with multiple hamartomas, and in 99% of cases, its initial presentation is mucocutaneous lesions. These precede, by a few years, the neoplasms most at risk for this syndrome such as renal cell neoplasia and thyroid and breast carcinoma [13] (Table 2).

**Table 2:** Neoplasic risk and age of onset

Tumor	Risk to life (%)	Beginning age
**Breast**	85	30
**Thyroid**	35	30-40
**Kidney cells**	34	50
**Endometrium**	28	40-50
**Gastrointestinal**	9	40
**Melanoma**	6	4

Adapted from https://rarediseases.org/rare-diseases/pten-hamartoma-tumor-syndrome/Copyright©2019

The importance of early diagnosis of this entity is related to the increase risk for certain cancers.

When the clinical phenotype raises suspicion, a screening test (https://www.lerner.ccf.org/gmi/ccscore/) can be carried out, which takes into account the individual’s age and phenotype. In adults, a clinical threshold score of ≥ 10 leads to a recommendation for referral to a genetics professional to consider PHTS. In children, macrocephaly and ≥ 1 of the following leads to the consideration of PHTS: (1) autism or developmental delay; (2) dermatologic features, including lipomas, trichilemmomas, oral papillomas, or penile freckling; (3) vascular features, such as arteriovenous malformations or hemangiomas; and (4) gastrointestinal polyps [13].

The diagnosis is mostly clinical, and the diagnostic criteria created by Eng have been developed and are reviewed annually by the National Comprehensive Cancer Network® (NCCN®) (Eng, 2000) (Table 3).

**Table 3:** Diagnostic criteria for Cowden syndrome

Criteria major	Criteria minor
*Breast cancer*	Colon cancer
*Follicular thyroid cancer*	Esophageal glycogenic acanthoses (≥ 3)
*Endometrial cancer*	Lipomas (≥ 3)
*Multiple gastrointestinal hamartomas*	Thyroid cancer (papillary or follicular variant of papillary)
Lhermitte-Duclos disease (adult)	Renal cell carcinoma
Macrocephaly (*P* ≥ 97)	Thyroid structural lesions (e.g., adenoma, nodule(s), goiter)
*Macular pigmentation of glans penis*	Intellectual disability (QI ≤ 75)
*Mucocutaneous lesions:* *● Trichilemmoma (≥ 3 biopsy proven)* *●* *Acral keratoses (≥ 3 palmoplantar keratotic pits and/or acral hyperkeratotic papules)* *● Oral papillomas (particularly on tongue and gingiva), multiple (≥ 3), or biopsy-proven or dermatologist-diagnosed mucocutaneous neuromas (≥ 3)*	Autism spectrum disorder
	Testicular lipomatosis
	Vascular anomalies (including multiple intracranial developmental venous anomalies)

Adapted from https://www.nccn.org Copyright©2019

Confirmation of the diagnosis of CS is confirmed when it presents (Pilarsky, 2019):

1. Three or more major criteria, but one must include macrocephaly, LDD, or gastrointestinal hamartomas; or
2. Two major and three minor criteria.

When there is a family history of CS or *PTEN* mutation, CS is considered in the individual presenting (Pilarsky, 2019):

- Two major criteria with or without minor criteria; or
- One major criterion and two minor criteria; or
- Three minor criteria.

The patient had several characteristic CS findings that satisfied three major criteria (macrocephaly, breast cancer, and mucocutaneous lesions) and two minor criteria (follicular variant of papillary thyroid cancer and intellectual disability), so she could be clinically diagnosed with CS.

NCCN recommends, for woman, an annual mammography of tomosynthesis starting at age 30–35 or 5–10 years before the earliest known breast cancer in the family. Given the 85% risk of developing breast cancer, the possibility of total mastectomy can be considered. Counseling should include a discussion regarding the degree of protection, reconstruction options, and risks. Because endometrial cancer can often be detected early based on symptoms, women should be educated regarding the importance of prompt reporting and evaluation of any abnormal uterine bleeding or postmenopausal bleeding. The evaluation of these symptoms should include endometrial biopsy every 1–2 years.

For both, women and men, NCCN advocates annual thyroid ultrasound starting at the time of CS/PHTS diagnosis, including childhood, and colonoscopy starting at age 35 years unless symptomatic or if the close relative with colon cancer before age 40, then start 5–10 years before the earliest known colon cancer in the family. Colonoscopy should be done every 5 years or more frequently if the patient is symptomatic or polyps are found. Renal ultrasound should be considered starting at age 40 and then every 1–2 years. Dermatological evaluation and treatment may be necessary for some patients. Children should have a thorough psychomotor assessment, and brain symptoms should be assessed with magnetic resonance imaging.

The differential diagnosis of entities related to PTEN hamartoma must be considered. Some characteristics of CS are similar to BRRS, such as macrocephaly, gastrointestinal hamartomas, cognitive impairment, and pigmented macules on the penis. The mutational prevalence in PTEN is around 60%; however, specific diagnostic criteria for this entity have not yet been crated. Regarding LDD, in which the prevalence of the affected PTEN is around 83%, the clinical findings are fundamentally based on slowly growing hamartoma of the cerebellum and usually diagnosed when patients are in their twenties or thirties [5].

When the trio of thyroid, breast, and renal cell carcinoma arises, not only the *PTEN* gene but also the *SDHB/C/D* gene should be screened [10].

The clinical evaluation and follow-up of these patients should be thorough so that the evolution of certain malignancies is detected in a timely manner and their diagnosis and treatment as appropriate as possible.

When diagnosed, patients should be instructed to be aware of the signs and symptoms of cancers with a higher incidence in this disease. Sirolimus is in phase II for PHTS and other drugs are being studied for solid neoplasms in individuals with CS that act on the pathway PI3K/akt/mTOR (BGT226 and BEZ235 in phase II).

## Data Availability

The datasets generated and analyzed during the current study are not publicly available due to belonging to the clinical entity (São João University Hospital Center) but are available from the corresponding author on reasonable request.

## Abbreviations

CS: Cowden’s syndrome
PTEN: Phosphatase and tension homolog
LLD: Lhermitte-Duclos disease
BRRS: Bannayan-Riley-Ruvalcaba syndrome
BIRADS: Breast Imaging-Reporting and Data System
